# Flexible workflows for on-the-fly electron-microscopy single-particle image processing using *Scipion*

**DOI:** 10.1107/S2059798319011860

**Published:** 2019-10-01

**Authors:** D. Maluenda, T. Majtner, P. Horvath, J. L. Vilas, A. Jiménez-Moreno, J. Mota, E. Ramírez-Aportela, R. Sánchez-García, P. Conesa, L. del Caño, Y. Rancel, Y. Fonseca, M. Martínez, G. Sharov, C.A. García, D. Strelak, R. Melero, R. Marabini, J. M. Carazo, C. O. S. Sorzano

**Affiliations:** a National Center for Biotechnology (CSIC), 28049 Cantoblanco, Madrid, Spain; b MRC Laboratory of Molecular Biology, Cambridge Biomedical Campus, England; c Universidad CEU San Pablo, Madrid, Spain; d Universidad Autónoma de Madrid, Madrid, Spain

**Keywords:** *Scipion*, image processing, electron microscopy, single-particle analysis, stream processing, electron-microscopy facilities

## Abstract

Electron microscopy of macromolecular structures is an approach that is in increasing demand in the field of structural biology. The automation of image acquisition has greatly increased the potential throughput of electron microscopy. Here, the focus is on the possibilities in *Scipion* to implement flexible and robust image-processing workflows that allow the electron-microscope operator and the user to monitor the quality of image acquisition, assessing very simple acquisition measures or obtaining a first estimate of the initial volume, or the data resolution and heterogeneity, without any need for programming skills. These workflows can implement intelligent automatic decisions and they can warn the user of possible acquisition failures. These concepts are illustrated by analysis of the well known 2.2 Å resolution β-galactosidase data set.

## Abstract 2

The *Scipion* framework allows very flexible image-processing workflows to be generated and employed at electron-microscopy facilities, such that image acquisition can be monitored and possible problems detected, thereby enabling early decisions to be made on the fly. The streaming workflow can be very simple or extended, permitting the data resolution and heterogeneity to be estimated and adapted to the desire of the user and the microscope operator.

## 1. Introduction   

Electron microscopy (EM) has become an established technique to define the three-dimensional structure of biological macromolecules (Frank, 2017 ▸). Owing to the high cost of the electron microscope itself, with all its components (direct electron detector camera, phase plates, spherical aberration correctors *etc.*), the current trend is to build large EM facilities that concentrate high-end machines and that offer their services to a large community of users. In such circumstances, it is advisable for the users to have previously screened the quality of their samples in more modest electron-microscopy setups.

During acquisition, the EM operator can monitor progress by watching the directories to which the movies are written, checking that new movies are effectively acquired and evaluating their quality (Alewijnse *et al.*, 2017 ▸; Gómez-Blanco *et al.*, 2018 ▸). Image processing of the new incoming movies is normally referred to as online, on-the-fly or stream processing, and there are several software suites to achieve this, such as *Appion* (Lander *et al.*, 2009 ▸), *Scipion* (de la Rosa-Trevín *et al.*, 2016 ▸), *Focus* (Biyani *et al.*, 2017 ▸), *RELION*-3 (Zivanov *et al.*, 2018 ▸), *SIMPLE* (Elmlund & Elmlund, 2012 ▸) and *Warp* (Tegunov & Cramer, 2018 ▸). The tools to process the streams have passed through several successive generations, enhancing their complexity and image-processing capacity.

(i) First generation. Movies are aligned and micrograph defocus is estimated. The shift parameters, power spectrum density of the micrograph and the defocus values are established and a thumbnail image is generated. Some statistical (mean, standard deviation and histogram) and time measurements (for example, a plot of defocus versus time) are summarized as acquisition progresses. Depending on the specific system, some thresholds may be applied to the maximum shift of a frame or movie. This was the status of, for example, *Scipion* v.1.1 and *Focus*.
(ii) Second generation. Particles can be automatically or semi-automatically identified in the electron micrographs, extracted and batch classified based on size. The user can manually inspect the 2D classes of the new batches and decide when problems occur in acquisition. This was the status of, for example, *Scipion* v.1.2.
(iii) Third generation. 2D classes are combined into an initial volume that may be further refined by structural refinement, assuming a homogeneous composition of the sample. This was the status of *RELION*-2.1 and *Appion*.
(iv) Fourth generation. Added intelligence and flexibility relative to the third-generation workflows. As such (1) the algorithm for a specific task is not ‘hardwired’ into a script but can be easily selected from a variety of options, (2) the image-processing pipeline can easily be tailored to the specific needs of a project or user, (3) several programs can be executed in parallel to perform the same task and a consensus output can be selected and (4) automatic decision algorithms also participate in the workflow so that different actions are taken depending on the quality of the micrograph and its particles. This is the status of *Scipion* v.2.0.

In this article, we show that *Scipion* v.2.0 allows image-processing pipelines to be constructed at any of these levels of complexity. The choice depends on the specific goal of the microscope operator, the needs of the microscope user and the computing capacities at the EM facility (although all of the image-processing workflows shown in this article require relatively simple hardware). The specific workflow shown here should not be taken as the ‘only’ possibility available in *Scipion*. In fact, this article focuses on single-particle analysis of structural proteins. However, specific but similar pipelines can be used for other sample types such as viruses, membrane proteins or helical proteins. The choice of certain packages and the parameters used depend on the experience of the user. *Scipion* currently allows any arbitrary workflow to be constructed as long as all of its components can run in ‘on-the-fly’ (streaming) mode, and most of the protocols now permit this up to particle extraction. Beyond particle extraction, only 2D classification using *Xmipp* (de la Rosa-Trevín *et al.*, 2013 ▸) is capable of working in full streaming mode at present. However, even static protocols (those that are not expected to be updated once they are started or those that cannot work in streaming mode) can be used to perfom specific tasks, producing static outputs.

In addition, since v.2.0 *Scipion* has provided smooth integration with more than 20 image-processing packages, each with an independent plugin. As such, these plugins can be updated, simplifying the fixing of bugs or the updating of features. Moreover, new plugins can be added for other packages or to provide new features such as beam-tilt assessment that are not yet available in *Scipion*. Note that all of these updates do not require an update of *Scipion*; rather, when a new plugin is found or a given plugin can be updated, it is simply highlighted in the plugin-manager GUI and can be easily installed or updated.

One of the advantages of having many different packages in the same platform is that it allows certain tasks to be executed with different algorithms in order to obtain consensus results. We find this to be very important because all algorithms fail at times and failures with one algorithm are not typically failures with another. In this way, consensus protocols are a useful way to automatically construct reliable results because these results are confirmed by different algorithms. Note that the execution of multiple algorithms for multiple tasks does not imply a combinatorial expansion of the analyses, requiring more computational resources. Rather, the consensus algorithm reduces this combinatorial expansion by combining the results of the different algorithms into a single output that can be further processed in the pipeline. In this way, the computational demands are suitably matched to relatively modest computers. Currently, *Scipion* can generate consensus results in the following three strategic steps: CTF estimation, particle picking and initial volume estimation.

In this paper, we have divided the processing stream into four logical steps according to the sequence of the data types at each point: from movies to micrographs, from micrographs to particles, from particles to 2D classes, and from 2D classes to the initial volume and an estimate of data resolution and heterogeneity. In the next four sections, we describe the possibilities of using *Scipion* for each of the four logical steps, illustrating its results for a particular workflow. Resources and time consumption are then evaluated in Section 6. Finally, Section 7 describe some examples of how to create and launch *Scipion* workflows to process data on the fly.

## 2. From movies to micrographs   

The electron microscope takes a collection of images of each field of view with very short exposure times; each image is called a frame. One of the key advances in the field was the realization that the sample was not static in space, but rather that it was moving (Brilot *et al.*, 2012 ▸). For this reason, frames must be aligned before they can be averaged into an electron micrograph. The signal-to-noise ratio (SNR) of these frames is extremely low (between 1/200 and 1/5000), such that the alignment algorithms must be extremely robust to noise and they must tolerate incorrect estimates of the alignment between any two frames. To perform this task, *Scipion* enables movie alignment while streaming through *Xmipp Correlation*, *Unblur* and *Summovie* (Campbell *et al.*, 2012 ▸; Grant & Grigorieff, 2015 ▸), *MotionCor*2 (Zheng *et al.*, 2017 ▸) and *Xmipp Optical flow alignment* (Abrishami *et al.*, 2015 ▸). We can consider these algorithms to be estimators of the deformation field between each of the frames and the final micrograph. In a way, we can assimilate the global alignment programs (*Xmipp Correlation* and *Unblur*) as Taylor zero-order estimates of these deformation fields. *MotionCor*2 allows parabolic deformation, which could be assimilated into a second-order estimate, and the *Xmipp optical flow* can be assimilated into a higher order estimate in which each pixel in the frames can move freely in any direction (with some regularization to ensure the smoothness of the deformation field). With the exception of *MotionCor*2, the programs have difficulties in following real-time processing using a single CPU, principally because their processing time may be longer than the acquisition time. However, there is no problem if multiple CPUs are available (depending on the data size, four or eight CPUs are normally sufficient), and this is certainly not a limitation if the alignment jobs are submitted to a cluster (for example, through queuing). *Scipion* streaming execution automatically handles the jobs that are finished and that are ready for the next step in processing.

Usually, users want to skip the first frames owing to the fast movement generated by sample charging and/or owing to beam-induced motion. For this reason, all movie-alignment protocols in *Scipion* are able to use a given range of frames. Owing to the high level of noise in the movies and the possible presence of artifacts, movie-alignment programs would be expected to make some errors from time to time. There is a protocol in *Scipion* that monitors the largest drift between two consecutive frames and the travel of the whole movie, such that if a frame within a movie or the whole movie moves more than a certain established threshold then the corresponding micrograph does not progress through the streaming pipeline and is set aside for subsequent inspection by the user. The use of this automatic selection protocol is optional in the workflow and illustrates the concept of adding some ‘intelligence’ (which is understood as making some automatic decisions depending on the quality of the data) to the image-processing pipeline. As such, objects that may be of dubious quality (either owing to the data itself or because of errors in the image-processing algorithms) are not fed blindly into the next image-processing step. Taking the 2.2 Å resolution β-galacto­sidase data set (Bartesaghi *et al.*, 2015 ▸) as an example, 2% of the movies were disabled by fixing thresholds of 5 Å within two consecutive frames and of 15 Å for the whole-movie drift when aligned by *MotionCor*2.

**Figure 1:**
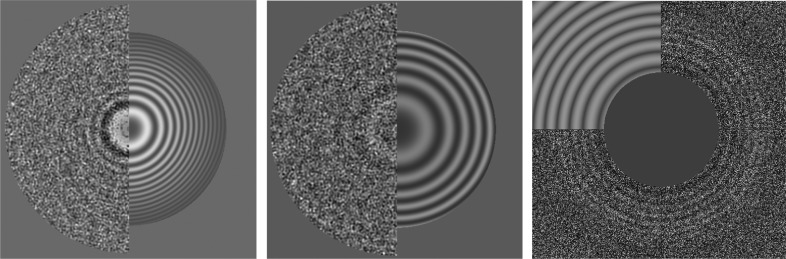
Example of automatic CTF selection for the 2.2 Å resolution β-galactosidase data set. Left: CTF disabled as *Xmipp CTF* estimates defocus U as 1.35 µm and defocus V as 1.21 µm, resulting in 0.14 µm of astigmatism in this case (less than 1% of the micrographs are disabled by this criterion). Centre: CTF disabled owing to poor visibility of the Thon rings (about 5% of the micrographs are disabled owing to this criterion). Right: CTF disabled owing to a large discrepancy between *Xmipp CTF* and *CTFFind*4 (about 5% of the micrograph are disabled owing to this criterion). After visual inspection, we realized that *CTFFind*4 has failed in this case.

Typically, the next step is to estimate the contrast transfer function (CTF) parameters, most importantly the defocusing. In streaming, *Scipion* offers *CTFFind* (Rohou & Grigorieff, 2015 ▸), *Gctf* (Zhang, 2016 ▸) and *Xmipp CTF* (Sorzano *et al.*, 2007 ▸; Vargas, Otón *et al.*, 2013 ▸) for this task. Many combinations can be established when using these programs; for instance, our workflow may use only one of them, two of them or all three in parallel (as independent estimates of de­focusing) or in a sequential mode (for instance, *Xmipp CTF* estimation provides a prior estimate of the defocusing as an initial value). *Xmipp CTF* has the advantage of calculating the CTF envelope, which is not estimated by *CTFFind* or *Gctf*. All of these programs are very fast and there is no problem in following the acquisition in real time. However, the defocusing of some of the micrographs is incorrectly estimated relatively frequently, either owing to a problem with their power spectra or because the estimation algorithm fails. There is an optional protocol in *Scipion* that calculates the consensus between two CTF estimations. Only those micrographs for which the defocusing and resolution values coincide between the two within a user-defined tolerance progress to the next stage, while the rest are set aside for subsequent inspection. Additionally, this protocol can also filter micrographs based on CTF quality criteria such as astigmatism, the visibility of Thon rings, the quality of the CTF fitting, the possible presence of aliasing, the presence of ice, maximum resolution, nonsensible phase-shift values, de­focusing range *etc.* (these types of thresholds are also available in *RELION*-3 and *Warp*). For the case of the 2.2 Å resolution β-galactosidase data set, 10% of the micrographs were filtered based on these quality criteria using the default thresholds or owing to discrepancies between *Xmipp CTF* and* CTFFind*4. Fig. 1 ▸ shows three examples of discarding CTF estimations based on different criteria.

Finally, we can produce a preprocessed set of micrographs in streaming by (i) eliminating hot spots (pixels whose values are clear outliers), (ii) inverting the contrast, (iii) performing a downsampling, (iv) cropping borders, (v) normalizing the micrographs and (vi) applying low-pass or high-pass filters (all of the steps or any combination of these can be employed). As always, this step is optional and *Scipion* can produce any number of these preprocessed sets of micrographs. For instance, we can produce a set in which only hot spots are eliminated (an important step to avoid artifacts around these points when some kind of CTF correction is performed), which is useful to extract the full-size particles, and another set with certain downsampling (to reduce the size of the micrographs) and a high-pass filter (to remove slow illumination gradients), which is useful for finding the particles and performing 2D class analysis while saving time and resources.

Over long acquisition periods (more than three days), we found that the camera gain degrades significantly for some reason (obviously depending on the microscope). *Scipion* includes another protocol that regularly estimates the camera gain every few hours in order to monitor its stability (Sorzano, Fernández-Giménez *et al.*, 2018 ▸).

**Figure 2:**
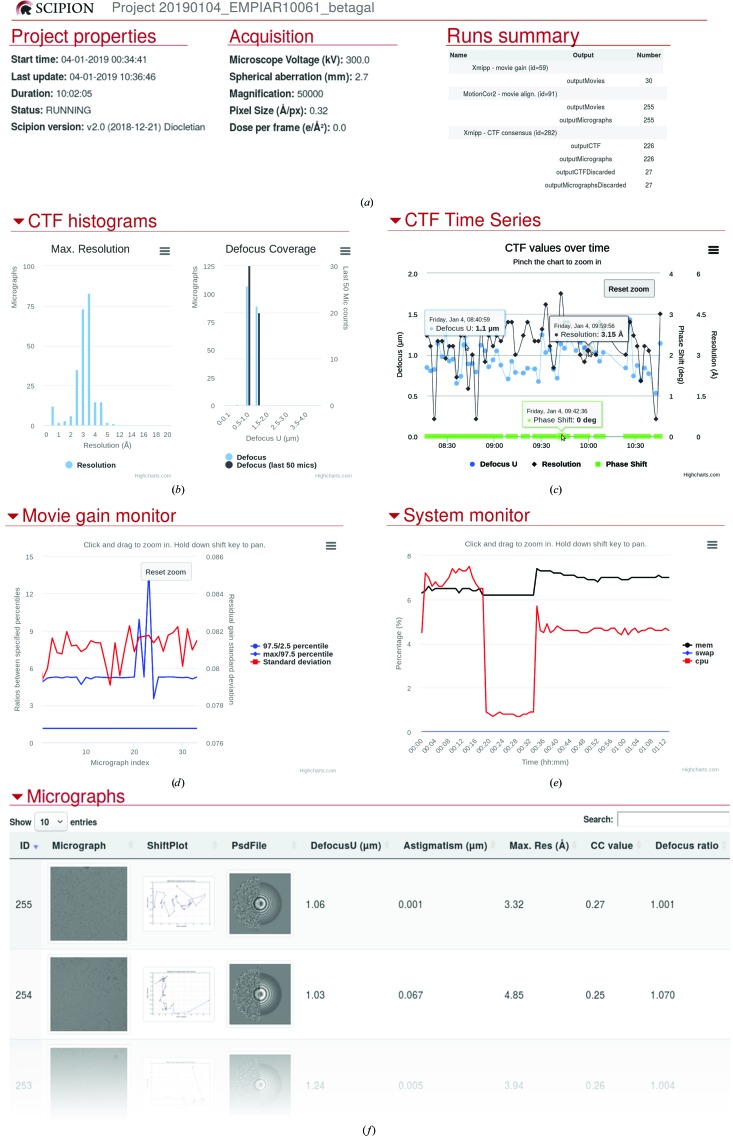
HTML summary of monitoring for the acquisition simulation of EMPIAR data set 10061. (*a*) Project summary, (*b*) the maximum resolution and defocusing histograms according to the CTF estimations, (*c*) CTF values (resolution, defocusing and phase) over time, (*d*) gain evolution, (*e*) system monitoring showing the CPU and RAM load and the swap over time and (*f*) the micrograph list showing the thumbnails of the aligned micrographs, the shift drift during the alignment, the estimated CTF and other estimated parameters, such as defocusing, astigmatism, maximum resolution, cross-correlation with the theoretical CTF and the astigmatism ratio.

From the point of view of the operator and user, it is important to have real-time feedback on the amount of data being acquired and on its quality in order to take timely decisions on the experiment being carried out. This is achieved in *Scipion* using a protocol that monitors all of the protocols described in this section and that generates an HTML report (see Fig. 2 ▸) that can be retained within the facility or published at any public location so that it can be accessed remotely from any device outside the institution (privacy and confidentiality are kept by simply sharing the public URL with the people involved in the specific project). In addition, this setup can be configured to send e-mail alerts to the microscope operator if the acquisition parameters (gain, defocusing, astigmatism or hardware availability) surpass any user-defined threshold. As such, the acquisition can be left unattended with a guarantee that the operator will be warned if something exceeds a given limit.

## 3. From micrographs to particles   

The stream processing described in the previous section used to be the only stream processing performed at the EM facility, and as such it can be regarded as a characterization and monitoring of the ‘functioning’ of the machine. At the end of the acquisition period, the user is given a report indicating the number of micrographs acquired and some statistics about the defocusing values, alignment shifts and expected resolution (from the CTF point of view). However, this analysis is not especially informative about the quality of the sample itself. To obtain a better sample analysis, image-processing packages now continue with the image processing of subsequent steps. Therefore, the next step is to find particles in the micrographs, which can be performed in four different ways using the programs available in *Scipion* v.2.0.

(i) By looking for objects of a given size [*SPARX* Gaussian picker (Hohn *et al.*, 2007 ▸), *RELION* Gaussian picking (Scheres, 2014 ▸) and *Appion* DoG picker (Voss *et al.*, 2009 ▸)].
(ii) By using a picker trained to select a variety of micrographs (*Sphire-crYOLO*; Wagner *et al.*, 2019 ▸).
(iii) By learning from the kind of particles to select (*Xmipp auto-picking*; Abrishami *et al.*, 2013 ▸).
(iv) By using templates to match areas in the micrographs, where these templates may come from a 2D analysis of the first micrographs in which the particles have been manually selected, may be selected by any other template-free picker or by generating projections from a structure similar to that under study (*Gautomatch* and *RELION reference-based picker*; Scheres, 2014 ▸).

These four families of algorithms have been sorted in an increasing order of the knowledge required about the specific structural analysis being performed. All of the previous pickers already work in streaming in *Scipion*, and while there are other packages such as *Warp* that can pick particles in streaming, *Scipion* does not yet support them. In contrast, other packages such as *boxer* in *EMAN*2 (Tang *et al.*, 2007 ▸; Woolford *et al.*, 2007 ▸) and *Bsoft* (Heymann & Belnap, 2007 ▸) are available in *Scipion* but not in streaming.

There are several key issues that have prevented the automation of particle picking in streaming to date.

(i) Except for the case of pickers based on templates from an external volume, all pickers must have information regarding the size of the particles studied. To date, this size could only be determined by opening one of the micrographs and inspecting the particle size, manually assigning this value to the picking protocols. In *Scipion* v.2.0, there is a new *Xmipp* protocol that can automatically assess the size of the particles, which is achieved by training a deep-learning algorithm with a variety of data sets of different sizes.
(ii) We can only ask for user intervention when there is sufficient information for him/her to intervene. For instance, if the user is required to train a picker, we can only launch the picker when there is a minimum number of micrographs that the user can inspect. This has been resolved in *Scipion* v.2.0 with the introduction of triggers that let the streaming flow pass only when a given number of images has been reached (micrographs in this case). The trigger has three operating modes: (1) dynamic (once the number of images is reached a single output is created and it is updated with new incoming data as soon as it is ready); (2) multiplexing (the incoming data are divided into different outputs with sizes at least equal to that of the threshold of images); and (3) static (once the threshold is reached the output only consists of the data that triggered the event and it is closed; this mode of operation is very useful to incorporate protocols that cannot run in on-the-fly mode into the image-analysis pipeline).
(iii) The need for user intervention (selecting the particle size or a few particles to train a picker) interrupts the streaming pipeline and the automatic execution of the subsequent steps cannot be started until the user intervention has ended. This has been resolved in *Scipion* v.2.0 by introducing a scheduling algorithm that automatically launches protocols only once all their inputs are ready, *i.e.* their parent protocols have started to produce an output.

**Figure 3:**
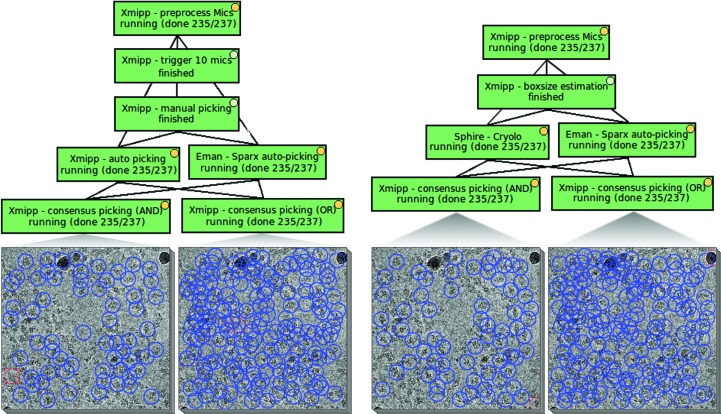
Particle-picking stage. Left: semi-automatic picking workflow, where manual picking trains the *Xmipp auto-picking* and fixes the particle size for *EMAN*2 *SPARX*. Right: fully automatic picking workflow using *EMAN*2 *SPARX* and *Sphire-crYOLO*, where the box size is estimated by *Xmipp*. The AND and OR consensus strategy is followed for both workflows and the resulting selections for certain micrographs from EMPIAR data set 10061, are shown.

Beside the wide variety of particle pickers available, *Scipion* also offers the possibility of running several of them in parallel (these algorithms are relatively fast and there is no problem in following the acquisition in real time) and of computing some kind of consensus between them. This is very useful because the number of false positives and false negatives for any particular picker can be non-negligible, and it may vary from as low as 5% to as high as 50% depending on the algorithm and the data set. With the consensus, we could run two or more pickers and trust the particles that were found by all pickers simultaneously within the tolerance defined by the user. This approach is very restrictive, but it is better that the particles selected are more likely to be true positives. We refer to this strategy as the AND strategy because a particle has to be found by algorithm 1 AND algorithm 2 AND algorithm 3 *etc.* At the other extreme, we could accept as a particle any coordinate suggested by any of the pickers. In this case we would be very loose in our criteria but it is much more likely that we will find all of the true particles present in the micrographs, although also many false positives. We refer to this strategy as the OR strategy because a particle is found by algorithm 1 OR algorithm 2 OR algorithm 3 *etc.* We can construct a consensus with any number of input pickers and with any number of coincident pickers for a coordinate that is considered a particle. At present, we have found that many users adopt the OR strategy in the hope that posterior image analysis will allow the incorrectly selected particles to be removed. However, both strategies make sense in a streaming pipeline, as with the AND strategy we can perform processing that produces results with a high degree of confidence that can then be used to clean the results from the OR strategy. See Fig. 3 ▸ for a comparison of the results from both strategies obtained using two different combinations of picking algorithms. At the end of the 2.2 Å resolution β-galactosidase session, around 100 000 particles were picked following the AND strategy after processing 1539 micrographs, whereas around 250 000 coordinates were considered as candidate particles using the OR strategy (the two workflows proposed in Fig. 3 ▸ have similar numbers). Note that the specific workflow implemented at each EM facility can be specified by the facility operator according to his/her past experience, computing capacity and personal preferences.

Particle extraction and normalization are available in streaming for *Xmipp* and *RELION*. At this point, it is customary to perform a phase-flip correction to compensate for changes of sign in the CTF. As performed previously, multiple particle extractions can be performed, for instance at different downsampling rates. One of the particle extractions can be used to perform a 2D class analysis and to identify incorrectly selected particles, while the other can be used to produce a set of full-sized particles. The subset of those particles identified as correct by the 2D analysis of the small images can also be selected in streaming from the set of full-sized particles. In addition to this standard processing, *Scipion* also allows other preprocessing steps to be performed on a streaming set of particles, such as cropping or resizing the particles, removing hot pixels, centering the images, inverting their contrast, thresholding them in multiple ways and applying low-pass and high-pass filters.

**Figure 4:**
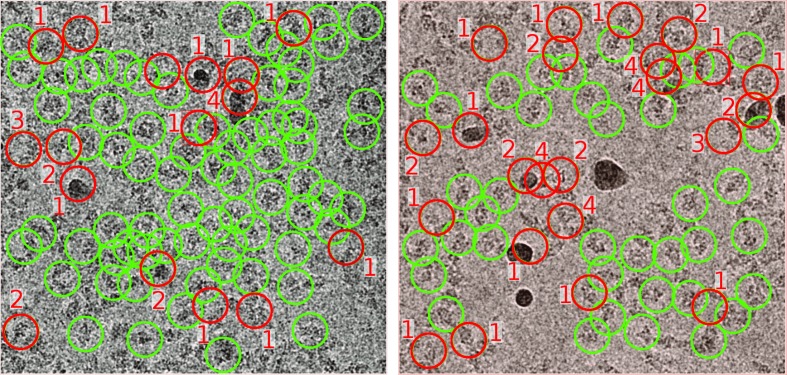
Automatic particle rejection for two micrographs from EMPIAR data set 10061. The red circles correspond to those particles that were labeled as incorrect, whereas the green circles correspond to those that were considered suitable to continue in the pipeline. The number beside each rejected particle corresponds to the reason why it was rejected (see Section 3).

At this point, relatively simple but very effective strategies can be used to get rid of incorrectly selected particles (Vargas, Abrishami *et al.*, 2013 ▸). These strategies are capable of removing ‘obviously’ incorrect particles, yet they are not able to identify small variations in the particles that impede high-resolution reconstructions being achieved. These strategies mostly come from *Xmipp* protocols working in streaming and they include (1) the elimination of empty particles (defined as those whose variance in the center of the image is not significantly larger than the surroundings), (2) the evaluation of the overall particle shape and gray values, discarding those that do not follow the general trend, (3) the elimination of those particles that are considered to be particularly noisy, either through analysis in real space or the frequency space, and (4) the elimination of particles whose context in the micrograph was considered to be abnormally variable (usually in regions of large aggregates, carbon edges or in the presence of contaminants or crystalline ice). Any combination of these filters can be used and the specific thresholds for each can be selected by the user. When we applied all of these filters (using the default thresholds) to the AND strategy on the 2.2 Å resolution β-galactosidase data set, 25% of the particles were rejected. This number may seem to be too large, but upon visual inspection of these particles we concluded that most of them were indeed false positives. Fig. 4 ▸ shows the discarded particles in red, with labels indicating the rejection criteria described above.

In the future, we intend to provide monitors that provide warnings if acquisition is taking place in a region that is giving a low particle yield or in which many of the particles selected are being rejected based on a measure of quality.

## 4. From particles to 2D classes   

Once we have a streaming set of particles, the standard image-processing workflow proceeds with the 2D classification to fulfill a series of objectives.

(i) To compress the set of several thousand projection images with a very low SNR into a comprehensible set of 2D averages with a higher SNR.
(ii) To identify incorrectly selected images [images characterized as different from the images belonging to the core or the stable core of the class (Sorzano *et al.*, 2014 ▸), or those assigned to a 2D class whose representative does not correspond to a centered projection of the structure under study (*i.e.* an artifact), that is in the middle of two particles or that corresponds to an empty region of the micrograph].
(iii) To evaluate the acquisition quality through the frequency content of the 2D averages (good acquisitions normally generate 2D classes with a very high frequency content, while acquisitions that are limited in their resolution for any reason, or that suffer image-alignment problems, produce poorly resolved 2D classes).
(iv) To evaluate the quality of the region currently being imaged.

We can follow different strategies to accomplish these objectives. The main 2D classifiers used within *Scipion* [*RELION* 2D (Scheres *et al.*, 2005 ▸) and *CL*2*D* (Sorzano *et al.*, 2010 ▸)] were not designed to work in streaming. We have expanded *CL*2*D* with a streaming version that runs on GPU and that has been specifically designed to follow real-time streaming. This extension can work in two modes: (i) fully dynamic 2D classification or (ii) semi-static 2D classification. In the first mode, the 2D class representatives and the images assigned to each 2D class are updated as new data come in. In the second mode, once a new image has been assigned to a certain class it remains in that class and is no longer updated. The second mode is much faster since the images already assigned are not reassigned. Still, the fact that *RELION* 2D and *CL*2*D* do not work in streaming does not prevent their use in on-the-fly processing, as we show below.

We have found four complementary strategies to be very useful.

**Figure 5:**
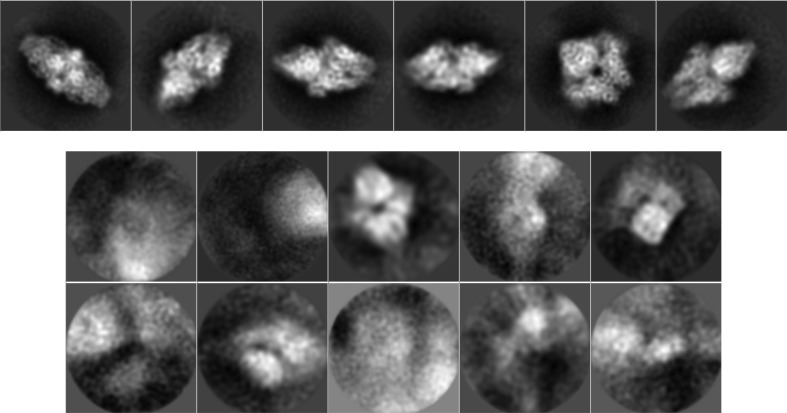
Automatic 2D class selection. Top: classes automatically selected for further processing. Bottom: classes automatically disabled as having either a heterogeneous background or representing very few particles.

(i) Creating a static 2D summary of the particles selected using the AND strategy (see Section 3), which serves to construct an initial volume based on the 2D classes found (see Section 5). This procedure can be performed once the AND set of particles reaches a given number of particles selected by the user (typically between 5000 and 10 000) using a trigger in the static mode (see Section 3). Once the trigger has been activated, its output is frozen at the desired number of particles so that the standard 2D classifiers (*RELION* 2D or *CL*2*D*) do not have any problem dealing with this input. We find that *CL*2*D* is capable of producing a larger variety of 2D classes than *RELION* 2D, and we prefer this option to draft the initial volume (typical numbers range from 16 to 64 classes). However, either of the two is possible, as is a conjunction of the output of both classifiers. In addition to the classification itself, we have implemented a *Scipion* protocol that automatically selects 2D class representatives that are more likely to represent good classes (employing the same algorithm used to detect empty particles described above), and the algorithm also takes into account the number of images assigned to that class (for an example of its results, see Fig. 5 ▸).
(ii) Creating a static 2D summary of the particles selected with the OR strategy. As above, this summary is activated by a trigger when the input set of particles has reached a given number of particles (typically between 10 000 and 20 000). For this summary, we found it useful to use *RELION* 2D and the 2D class representatives of the AND strategy. *RELION* 2D is very good at producing classes that attract empty or incorrectly selected particles, while the summary from the AND picking strategy contains a good representation of the 2D variability present in the data set. In general, the set of classes from this summary ranges from 30 to 100 and it contains 2D representatives corresponding to projections of the structure under study, as well as 2D representatives corresponding to consistent artifacts, empty regions *etc.*
(iii) The static 2D summary constructed is used in the semi-static 2D classification described above. In this way, we classify the stream of particles coming from the OR picking strategy into one of the possible classes available. This classification is very fast since the 2D class representatives are not updated and the stream can be followed in real time without any problem. On terminating the acquisition, when the stream is closed because no more particles enter, we simply need to create a subset with all of those particles assigned to the class of interest and leave out all of the images assigned to un­interesting classes. For the case of the 2.2 Å resolution β-galactosidase data set, 80% of the particles from the OR strategy were assigned to one of these interesting classes (about 150 000 particles remained) and this was the final set of particles from the stream processing to be used for the detailed processing to obtain the high-resolution volume.
(iv) To evaluate the current acquisition region, the following strategy proved to be of interest. The input stream set is divided into small batches of particles whose size ranges from 5000 to 20 000. These small sets are static (once created they do not keep track of the stream) and they are classified using any of the existing 2D classification methods. The output of each one of the classifications can be manually inspected to evaluate their quality, and if their quality is unsatisfactory then acquisition can be shifted to any other region of the grid or to a different grid. Although not implemented at present, we plan to automate this process in the future so that a warning can be given if the acquisition is currently in a region with 2D alignment and/or classification problems (probably owing to a heterogeneous ice layer, the presence of some contaminant or problems with the support).

## 5. From 2D classes to initial volume, and an estimate of data resolution and heterogeneity   

**Figure 6:**
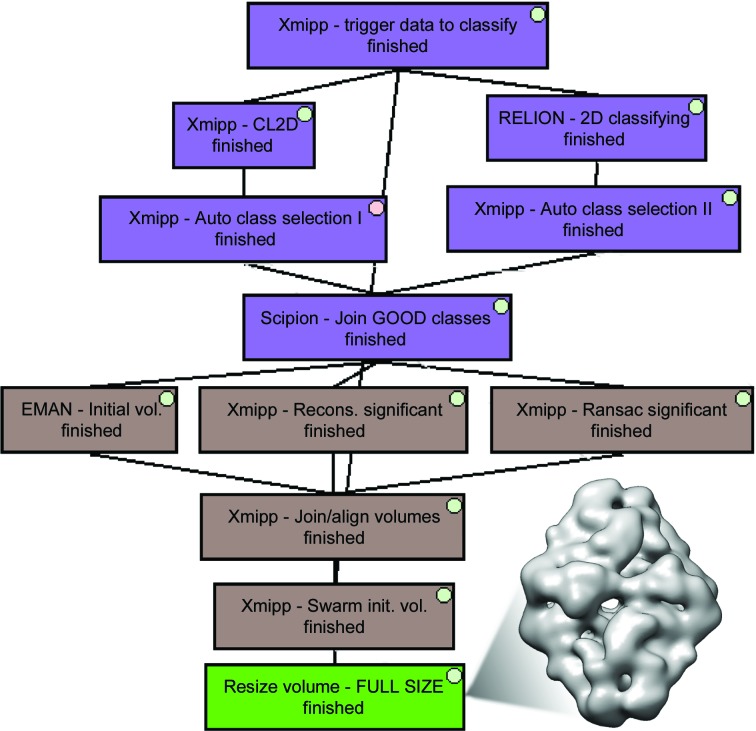
Workflow example of 2D classification (purple boxes) and initial volume reconstruction (brown boxes). Since the 2D analysis is carried out on downsampled images, the resulting initial volume is resized to the original size (green box). The volume shown at the side of the workflow corresponds to the classification of 5000 particles from the 2.2 Å resolution β-galactosidase data set, which results in 17 automatically selected classes from a total of 32 (16 for *CL*2*D* and 16 for *RELION* 2D). In addition, *Xmipp Swarm consensus* has merged 21 initial volumes (ten from *EMAN*, ten from *Xmipp Ransac* and one from *Xmipp Significant*).

In some projects, the initial volume is known from the very beginning, before any image processing is performed (for instance, when studying the structure of a macromolecule bound to a ligand if the structure of the macromolecule without the ligand is already known). However, in many other projects this initial volume is not known, or constructing the initial volume from the data itself serves as a validation of any prior assumptions. The construction of this volume does not need to be performed in streaming, as an initial volume for this study can be calculated once a given number of particles has been reached (typically between 5000 and 10 000). *Scipion* offers several algorithms for this: *EMAN*, *Xmipp Ransac* (Vargas *et al.*, 2014 ▸), *Xmipp Significant* (Sorzano *et al.*, 2015 ▸), *RELION* (Scheres, 2016 ▸) and *Simple Prime*3*D* (Elmlund *et al.*, 2013 ▸). They normally work on class averages, although some of them can also work on a set of particles. These algorithms produce one or several candidates for the initial volume, and the number of incorrect initial volumes depends on the particular specimen, although it may be non-negligible. Typically the user has to choose one of them as the initial volume to continue the study, and this choice can represent an important bias in the overall analysis. Recently, we introduced an algorithm called *Xmipp Swarm consensus* that can automatically calculate a consensus of initial volumes (Sorzano, Vargas *et al.*, 2018 ▸) from a set of initial volume proposals and a set of particles. In this way, the selection is less biased. In the example that illustrates the streaming capacities of *Scipion* shown here, we used *EMAN*, *Xmipp Ransac* and *Xmipp Significant*, combining them into a single volume using *Xmipp Swarm consensus* (see Fig. 6 ▸).

Once the initial volume has been constructed, we can use it to estimate the resolution and heterogeneity of the data. As such, another static trigger is launched on the AND particle selection branch, triggered at 30 000 particles, even though any other number could have been used. This trigger launches particle refinement (in particular *RELION* *autorefine* owing to its speed) and 3D classification (also with *RELION* for reasons of speed). For the β-galactosidase example, the resolution of *autorefine* reached 3.6 Å. Note that this resolution is lower than that reported in EMPIAR, yet we must take into account that we have only obtained it in a fully automatic way with the first 30 000 particles. In any case, this step is useful to check whether the acquisition is proceeding satisfactorily and also to obtain an idea of the expected resolution when all of the particles are processed. The 3D classification into three classes produced two similar classes with 64% of the images and a junk class with 36% of the images (incorrectly identified by the picking algorithms). This analysis indicated that the micrographs did not contain different populations of macromolecules, yet the 3D classification should be able to separate any larger heterogeneity in the sample if it exists.

## 6. Resources and time consumption   

Most of the resources and time-consuming protocols in the full streaming process reside in the first steps of the workflow, especially in the movie-alignment algorithms, followed by the CTF estimators and the pickers (Table 1 ▸). The rest of the protocols until the 2D classification can seamlessly follow the acquisition rate because the vast majority of the tasks involve dealing with the databases or downsampled images. Once in the 2D classification, the work involves batches of data, and thus the performance can be adjusted by fitting to the sizes of the batches.

## 7. Creating and launching stream workflows   

Each EM facility and/or user may use a customized streaming image-processing workflow. On a more basic level, *Scipion* accepts two possible ways of creating and launching these workflows.

**Figure 7:**
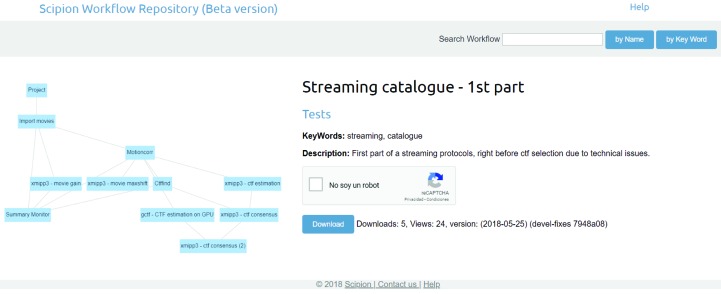
Example of a workflow downloaded from the public repository http://workflows.scipion.i2pc.es.

(i) Use the *Scipion* API to create an empty project and then create the image-processing pipeline by adding new objects to the protocol, linking their inputs and outputs as required. This option requires Python programming skills, although it is very flexible as the user (programmer) has all the protocols to hand and is completely free to create any possible workflows.
(ii) Creating a workflow from a workflow template. These can be created by exporting an existing workflow as a JSON file, which is then imported and scheduled for execution. There is a repository of publicly available workflows at http://workflows.scipion.i2pc.es that can be downloaded and used at will (see Fig. 7 ▸).

In either of these two cases, new image-processing steps (boxes) can be added from the *Scipion* GUI. Alternatively, the whole streaming workflow can be created manually from the GUI by adding all of the required steps. However, this is less convenient for production facilities where there is only one or just a few standardized image-processing pipelines.

**Figure 8:**
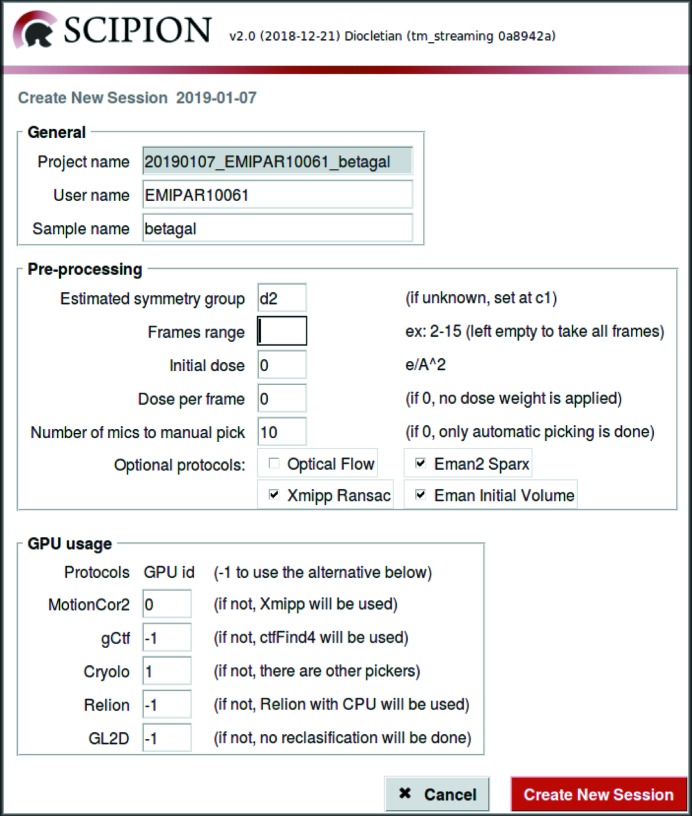
Example of a high-level tool that can create a configurable image-processing workflow in streaming. The tool gives a choice between different algorithmic alternatives for each of the steps and of the configuration of the hardware (in particular GPU and CPU) usage.

These two low-level access points can be more conveniently used from high-level tools. For instance, we can configure the *Scipion* API by creating an application that writes the Python script to describe the workflow. An example of this is shown in Fig. 8 ▸.

**Figure 9:**
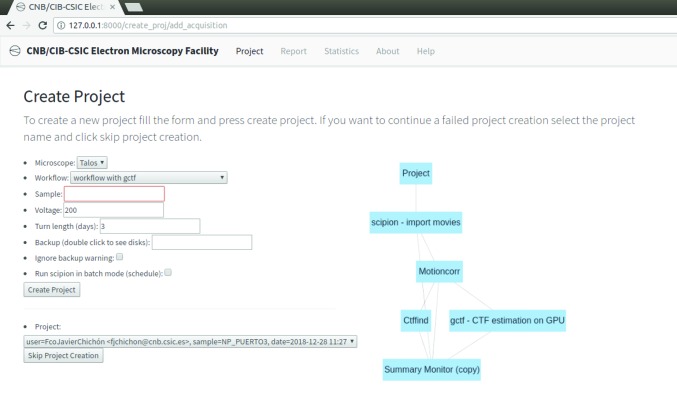
Example of a high-level web tool that can import an existing processing streaming workflow.

Alternatively, we may design a web page that automatically creates the project, imports the JSON workflow template from a set of possibilities and launches it. This is the solution that we have adopted at the National Center of Biotechnology (CSIC) for our own EM facility (see Fig. 9 ▸).

Finally, *Scipion* also offers a very simple but flexible option that consists of modifying some fields of an existing JSON template (created as indicated above), using an easy syntax to launch a workflow similar to that shown in Fig. 8 ▸, which can then be used as a starting point for data processing.

More specific information on how to prepare streaming workflows can be found on the documentation page at http://scipion.i2pc.es.

## 8. Conclusions   

In this article, we have presented the possibilities that *Scipion* offers to design on-the-fly image-processing workflows. We illustrate these possibilities with a particular workflow, yet simpler or more complex workflows could have been designed and deployed at any EM facility. The customization of the workflow may depend on the computing capabilities available, the goal of the analysis (characterizing the microscopy session or the sample), the experience of the EM operator and user *etc*. *Scipion* supports a wide variety of algorithms for each task and although not all of them can be used in streaming, as shown here, this does not prevent them from participating in useful streaming workflows.

The current implementations of the 3D classification and reconstruction algorithms have not been designed with streaming processing in mind. However, nothing conceptually prevents them from managing a streaming data flow and more advances in this regard should be expected in the future.

*Scipion* is a project manager that places special emphasis on the traceability and reproducibility of the image-processing workflow. This is an absolute requirement in an EM facility, not only to be able to monitor every acquisition in a controlled scope, but also to be able to export the processed data in self-contained projects in which all the data, metadata, parameters and operations have been registered. This enables all of the work performed during the acquisition process to be used as the starting point for user-assisted refinement processing, simply by taking the project directory from one computer to another and opening the project in *Scipion*.

Additionally, the availability of several algorithms to perform the same task allows consensus results to be constructed, which are much more reliable than using a single algorithm, and we show how these consensus algorithms can participate in streaming workflows. Finally, we have also shown how to incorporate monitoring protocols that can alert the EM operator to specific situations so that early decisions can be taken with the user to optimize the outcome of the microscopy session.

*Scipion* v.2.0 has been ‘pluginized’, meaning that all software updates from the underlying packages that perform the image processing can automatically be made available to the user as soon as the plugin is updated.

## Figures and Tables

**Table 1:** Algorithm benchmarks The movies to align are made up of 7676 pixels × 7420 pixels × 38 frames (0.32 Å per pixel) and the original sizes of the micrographs are 7676 pixels × 7420 pixels (0.32 Å per pixel), while the downsampled micrographs are 1228 pixels × 1187 pixels (2.0 Å per pixel) and the downsampled particles are 110 pixels × 110 pixels (2.0 Å per pixel). The CPUs used were Intel Xeon CPU E5-2630 v.4 (2.20 GHz) and the GPUs were Nvidia GeForce GTX 1070. All tests were run on an SSD disk Micron M510DC 6 Gb s^−1^ SATA-2.5.

Algorithm	Time per step	Step	Comments
*MotionCor*2	1 min	Movie (0.32 Å per pixel)	9 × 9 patches, GPU
*Xmipp* *MotionCorB*	1 min	Movie (0.32 Å per pixel)	10 × 10 patches, GPU
*Xmipp CTF*	20 s	Micrograph (0.32 Å per pixel)	CPU
*CTFFind*4	30 s	Micrograph (0.32 Å per pixel)	CPU
*Gctf*	4 s	Micrograph (0.32 Å per pixel)	GPU
*Xmipp auto-picking*	0.5 s	Micrograph (2.0 Å per pixel)	User training on the fly, CPU
*Sphire-crYOLO*	0.5 s	Micrograph (2.0 Å per pixel)	General pre-trained model, GPU
*EMAN*2 *SPARX*	0.7 s	Micrograph (2.0 Å per pixel)	CPU
*Xmipp* extract particle	1 s	Micrograph (2.0 Å per pixel)	Applying the phase flip, CPU
*Xmipp* *CL*2*D*	20 min	5000 particles (2.0 Å per pixel)	8 classes, GPU
*RELION* 2D classification	13 min	5000 particles (2.0 Å per pixel)	8 classes, GPU
*Xmipp Ransac*	4 min	17 class averages (2.0 Å per pixel)	8 × CPU
*Xmipp Significant*	28 min	17 class averages (2.0 Å per pixel)	32 × CPU
*EMAN*2 initial volume	2 min	17 class averages (2.0 Å per pixel)	8 × CPU
*Xmipp Swarm consensus*	2 h 51 min	5000 particles (2.0 Å per pixel)	32 × CPU
*RELION* 3D classification	1 h 16 min	5000 particles (2.0 Å per pixel)	3 classes, GPU
